# Nutritional Screening and Anthropometry in Patients Admitted From the Emergency Department

**DOI:** 10.3389/fnut.2022.816167

**Published:** 2022-02-14

**Authors:** Enza Speranza, Lidia Santarpia, Maurizio Marra, Olivia Di Vincenzo, Marianna Naccarato, Carmela De Caprio, Delia Morlino, Gaetano D'Onofrio, Franco Contaldo, Fabrizio Pasanisi

**Affiliations:** Internal Medicine and Clinical Nutrition Unit, Department of Clinical Medicine and Surgery, Federico II University Hospital, Naples, Italy

**Keywords:** Hospital malnutrition, anthropometry measurements, malnutrition risk, Subjective Global Assessment (SGA), Nutritional Risk Screening (NRS 2002), GLIM criteria

## Abstract

**Background:**

Due to the high prevalence of malnutrition among hospitalized patients, screening and assessment of nutritional status should be routinely performed upon hospital admission. The main objective of this observational study was to evaluate the prevalence of and the risk for malnutrition, as identified by using three nutritional screening tests, and to observe whether some anthropometric and functional parameters used for nutritional evaluation were related to these test scores.

**Methods:**

This single-center observational study included 207 patients admitted from the emergency department for hospitalization in either the internal medicine or surgery units of our institution from September 2017 to December 2018. The prevalence of malnutrition among this patient sample was evaluated by using the Nutritional Risk Screening (NRS-2002), the Subjective Global Assessment (SGA) and the Global Leadership Initiative on Malnutrition (GLIM) criteria. Body mass index (BMI), bioimpedance analysis (BIA), handgrip strength (HGS) and calf circumference (CC) assessments were also performed.

**Results:**

According to the NRS-2002, 93% of the patients were at no risk or at low nutritional risk (NRS score < 3), and 7% were at a high nutritional risk (NRS score ≥ 3). On the other hand, according to the SGA, 46.3% of the patients were well-nourished (SGA-a), 49.8% were moderately malnourished (SGA-b), and 3.9% were severely malnourished (SGA-c). Finally, according to the GLIM criteria, 18% patients were malnourished. Body weight, body mass index (BMI), phase angle (PhA), CC and HGS were significantly lower in the patients with NRS scores ≥ 3, SGA-c and in patients with stage 1 and stage 2 malnutrition, according to the GLIM criteria.

**Conclusion:**

The NRS-2002, the SGA and the GLIM criteria appear to be valuable tools for the screening and assessment of nutritional status. In particular, the lowest NRS-2002, SGA and GLIM scores were associated with the lowest PhA and CC. Nevertheless, a weekly re-evaluation of patients with better screening and assessment scores is recommended to facilitate early detection of changes in nutritional status.

## Introduction

Disease-related malnutrition among hospitalized patients is a major public health issue in both industrialized and developing countries around the world, with a reported prevalence between 20 and and 50%, according to differences in study populations, assessment methods, and hospital settings (1, 2). Malnutrition due to starvation, disease or aging can be defined as “a state resulting from lack of uptake or intake of nutrition with altered body composition (such as decreased fat-free mass) and body cell mass leading to reduced physical and mental functioning and poor clinical outcomes” (3). Poor nutritional status is associated with a high disease burden, a large number of comorbidities and significant economic costs (4).

To identify patients who are at risk for malnutrition or who are malnourished upon hospital admission, nutritional screening should be performed as a part of standard care. Screening tests must be quick and easy to apply; in already malnourished patients, a thorough nutritional assessment should also be performed (2, 3).

The European Society for Clinical Nutrition and Metabolism (ESPEN) recommends using the Nutritional Risk Screening 2002 (NRS 2002) to screen adults upon hospital admission (5).

The Subjective Global Assessment (SGA) is a nutrition assessment test used worldwide that allows for the grading of nutritional status in different conditions; it includes a more complex set of questions and must be conducted by specially trained professionals.

Objective methods for nutritional assessment include anthropometry (BMI and CC, among others) body composition evaluated by BIA and functional tests.

In literature, several studies have already evaluated the relation between parameters predicting malnutrition and poor clinical outcomes (6). The prevalence of nutritional risk, and the consequences of malnutrition on patient outcomes in hospital setting (7, 8).

Regarding BMI, values lower than 18.5 kg/m^2^ suggest chronic malnutrition and are associated with poor outcomes and high mortality rates (9). Unfortunately, BMI may often be biased by fluid overload and oedema; moreover, due to the obesity pandemic, patients classified as malnourished may also have BMI values still in the normal range or even in the overweight or obese categories (10).

Low values of calf circumference (CC) seem to be associated with malnutrition or high nutritional risk (11, 12).

Bioelectrical impedance analysis (BIA) is a non-invasive, low-cost and simple method widely used to assess body composition; BIA-derived phase angle (PhA) is a reliable indicator commonly used for nutritional assessments (13, 14). It is inversely correlated with disease severity, inflammation and malnutrition in several clinical conditions (15, 16).

Finally, handgrip strength (HGS) is a validated and easily implemented measure of muscle strength that has been frequently used for clinical purposes, particularly in recent years. Low HGS values are associated with long hospitalization durations and high re-admission rates (17, 18).

In order to standardize the diagnosis of malnutrition, the Global Leadership Initiative on Malnutrition (GLIM) criteria, were also applied. The GLIM criteria are now considered as the best ones for diagnosing malnutrition in adults in clinical care settings and are based on a two-step model for the risk screening and diagnostic assessment of malnutrition (19).

Anyway, different screening tools may lead to different results for the risk of malnutrition, due to their different clinical significance (20). No research has been published that focused on how to choose the optimal screening tool.

Our study aims to evaluate the prevalence of and the risk for malnutrition, as determined by the NRS-2002 screening test, the SGA and by GLIM criteria, in patients upon hospital admission from an emergency department and in hospitalized patients in the internal medicine unit and the surgery unit of a single center and, to observe whether some specific parameters used for nutritional assessment were related to NRS-2002 and SGA test scores.

## Subjects And Methods

The sample size was calculated considering the primary endpoint and assuming, according to the literature (6, 7), an estimated proportion of malnutrition of 30% and a confidence level of 0.5. The required sample size was 234, and allowing for a 15% dropout rate, a final sample size of 207 patients was determined.

All Caucasian adult patients coming from the emergency unit and admitted to either the internal medicine unit or the surgery unit were recruited in our study from September 2017 to December 2018.

All patients were consecutively screened for inclusion.

The exclusion criteria were transfer from an intensive or critical care unit, pregnancy or breastfeeding, and inability to communicate.

Fifty patients refused to participate, 80 presented exclusion criteria, and 30 were excluded for other reasons; finally, 207 patients were eligible for inclusion in the study.

Informed consent was obtained just after admission, and the tests were performed during the first 48 h after admission by the same staff members to reduce the risk of bias during the measurement process. The study staff members included three dietitians and a biologist with expertise in clinical nutrition; they were all trained, according to the good clinical practice guidelines, with theoretical and practical sessions, to perform the screening tests and the measurements.

All data were collected and stored following SQL Server Security Best Practices.

### Nutritional Risk Screening 2002 (NRS 2002)

The NRS-2002 takes into account weight loss, BMI, food intake reductions and impaired general conditions. The disease severity score considers current clinical conditions as well as chronic diseases with acute complications (major abdominal surgery, stroke, head injury, or bone marrow transplantation). Scores from 0 to 3 correspond to these conditions. The total score is obtained from the nutritional and disease severity evaluation and adjusted for age in patients older than 70 years (+ 1 point), according to Kondrup's recommendations (5, 21). An NRS score < 3 indicates no risk for malnutrition, an NRS score ≥3 indicates a high risk for or clear malnutrition and is an indication of the need for nutritional support. The NRS-2002 has been assessed and validated in several studies, including randomized controlled trials, and has been shown to be reliable when administered by trained staff (22).

### Subjective Global Assessment (SGA)

The SGA includes the patient's history (weight loss, changes in food intake habits, gastrointestinal symptoms and functional capacity), a brief physical examination (checking for muscle wasting, subcutaneous fat, ankle and sacral oedema, and ascites) and the clinician's overall evaluation of the patient's status. Each patient is classified as well-nourished (SGA-a), suspected or moderately malnourished (SGA-b), or severely malnourished (SGA-c) (23).

### GLIM Criteria

The GLIM criteria are composed of three phenotypic and two etiologic criteria. A combination of at least one phenotypic (unintentional weight loss >5% within the past 6 months, low BMI and reduced muscle mass), and one etiologic (reduced food intake/ assimilation and disease burden) criterion is necessary to make diagnosis of malnutrition.

In our study, Based on the available data, the GLIM phenotypic criteria used for diagnosis of (a) moderate malnutrition or (b) severe malnutrition were also employed: (1) % weight loss: (a) > 5% or (b) > 10% and; (2) BMI: (a) if age <70 and BMI at admission <20 kg/m^2^ or age ≥70 and BMI at admission <22 kg/m^2^; (b) age <70 and admission BMI <18.5 kg/m^2^ or age ≥70 and admission BMI <20 kg/m^2^ (19).

Body composition measurements to assess muscle mass deficit, for the etiologic GLIM criterion the following criteria were used based on data that were available: (1) fat free mass index (FFMI, kg/m^2^) (2) presence of inflammation acute disease/injury related, another GLIM phenotypic criterion, because all patients coming from an Emergency Department.

### Anthropometry

Weight and height were measured or derived from indirect measures. Weight loss was generally self-reported by the patient. When the height of the patients was not assessed in the standing position (e.g., because they were bedridden, immobilized, or had just undergone surgery), specific formulas that involved knee height were used (24).

BMI was calculated as weight/height^2^ and was divided into the following categories:

BMI < 18.5 kg/m^2^ indicated underweight; 18.5 ≤ BMI > 25 kg/m^2^ indicated normal weight; and BMI ≥25 kg/m^2^ indicated overweight—obesity (25).

CC was measured by using an anelastic tape at the largest circumference; a CC of < 31 cm was considered a marker for muscle mass loss in older adults (11, 12).

### Handgrip Strength

Handgrip strength (HGS) was measured by using a dynamometer (JAMAR, Roylan, UK).

Bedridden subjects moved their arms parallel to the trunk, grasped the dynamometer, and applied the maximum force possible with each hand.

The measurement was repeated three times, in 1-min intervals to avoid fatigue, with each hand or on one side only in patients undergoing intravenous therapy and in those with other limitations; the mean and the maximum of the three recorded measurements were recorded in kilograms (kg).

The cut-off points used to diagnose dynapenia were < 27 kg for men and < 16 for women (26).

### Body Composition Measurements

Bioimpedance analysis (BIA) was performed using a Human Im Touch device (DS Medica srl, Milan, Italy) that measures resistance, impedance and PhA at 50 kHz. With the subject lying supine, four surface electrodes were placed on the non-dominant wrist and ankle. The patients were tested in their rooms after at least 15 min of lying during the first 48 h post hospital admission.

BIA takes into account resistance and the phase angle. Body composition parameters, such as fat-free mass (FFM) and fat mass (FM), were assessed by the BI-Index (height in cm^2^)/resistance ohm) using the Sun BIA equation (27). We used a cut-off value of 5° for the phase angle in both females and males because, in recent studies, PhA values < 5° have been shown to be associated with frailty and clinically adverse outcomes, such as incident disability and mortality, and are considered predictors of survival in several pathological conditions. It has also been demonstrated that decreases in different forms of malnutrition are associated with increased nutritional risk in various groups of patients (27–29).

### Statistical Analysis

Continuous variables are expressed as the mean values ± standard deviation (Mean ± SD).

Student's *t* test for unpaired data was used to evaluate the differences among groups with different test scores, and the chi-squared test was used to evaluate the relative frequencies within different test score groups. A normality test for variables has been performed, where applicable, and statistical analysis done accordingly. The Man-Whitney test was used for non-parametric variables.

All reported *p* values are based on two-sided tests and compared data among different groups to a significance level of 5%.

Independent associations among variables were assessed with stepwise regression analysis.

Statistical analysis was performed using SPSS for Windows, version 27.0 (SPSS Inc., Chicago, IL).

## Results

Our study included 207 Caucasian patients, 110 (53%) males and 97 (47%) females, aged between 18 and 85 years (60.3% *n* = 125 were older than 60 years), with a BMI range of 26.2 ± 5.4 kg/m^2^ (6% *n* = 12 had a BMI <18.5, and 22.2% *n* = 46 had a BMI higher than 30 kg/m^2^). Fifty-five percent (*n* = 114) of the patients was hospitalized for a cardiovascular or hepatic impairment, 23% (*n* = 48) for a diagnosis of oncologic disease and 22% (*n* = 46) for a gastrointestinal disease.

The anthropometric and body composition characteristics of the study participants are shown in Table 1. Age, weight and BMI were significantly higher among males than among females. HGS was lower among females, while no sex differences were observed for PhA or calf circumference.

**Table 1:** Anthropometric characteristics, body composition and handgrip strength in 207 patients.

	**Males** **(*n* = 110)**	**Females** **(*n* = 97)**	**Total sample** **(*n* = 207)**
**Age (years)**	64.9 ± 15.3^*^	59.5 ± 17.9	62.3 ± 16.7
**Weight (kg)**	75.4 ± 15.9^*^	68.3 ± 16.0	72.1 ± 16.3
**Stature (cm)**	171 ± 7.7^*^	160 ± 5.8	166 ± 8.8
**BMI (kg/m** ^**2**^ **)**	25.7 ± 4.6^*^	26.8 ± 6.2	26.2 ± 5.4
**Calf circumference (cm)**	35.2 ± 4.4	35.3 ± 4.4	35.2 ± 4.4
**Phase angle (degrees)**	4.8 ± 1.4	4.5 ± 0.9	4.7 ± 1.2
**HGS (kg)**	24.0 ± 9.4^*^	14.0 ± 5.2	19.3 ± 9.2

* *p < 0.005 between sexes. BMI, body mass index; HGS, handgrip strength*.

According to the NRS-2002, 193 (93%) patients were at no risk or at low nutritional risk, and 14 (7%) were at high nutritional risk. On the other hand, the SGA assessed those 96 (46.3%) patients were well-nourished, 103 (49.8%) were moderately malnourished, and 8 (3.9%) were severely malnourished.

According to the GLIM criteria, 37 (18%) patients were malnourished; in particular 18 patients had moderate malnutrition (stage 1) and 19 patients severe malnutrition (stage 2) (Table 2).

**Table 2:** Anthropometric characteristics, handgrip strength and body composition according to the GLIM criteria (stage 1 = moderate malnutrition; stage 2 = severe malnutrition).

	**Stage 1** **(*n* = 18)**	**Stage 2** **(*n* = 19)**	**Not malnourished patients** **(*n* = 188)**
**Age (years)**	67.1 ± 14.6	64.8 ± 16.8	61.6 ± 17.1
**Weight (kg)**	68.1 ± 18.7	70.3 ± 16.8	72.8 ± 16.1
**Stature (cm)**	166.8 ± 9.1	164.6 ± 7.9	165.6 ± 8.8
**BMI (kg/m** ^**2**^ **)**	24.3 ± 5.7	25.6 ± 4.7	26.5 ± 5.5
**Calf circumference (cm)**	33.2 ± 4.7	34.4 ± 4.9	35.6 ± 4.1^**^
**Phase angle (degrees)**	3.6 ± 0.9	3.8 ± 0.9	4.9 ± 1.2^*^
**HGS (kg)**	17.6 ± 8.1	16.3 ± 7.0	19.8 ± 9.4

* *p < 0.005 vs. Stage 1 and Stage 2; BMI, body mass index; HGS, handgrip strength*.

** *p < 0.005 vs. Stage 2*.

The patients with NRS scores ≥3 (4 M; 10 F) were older and had lower body weight, and BMI than those with NRS scores < 3 (100 M; 93 F); moreover, they had lower CC, HGS and PhA than those patients with NRS scores <3 (Table 3).

**Table 3:** Anthropometric characteristics, body composition and handgrip strength according to NRS score in males, females and total sample.

	**NRS** **< 3**			**NRS** **≥ 3**		
	**M** **(n. 100)**	**F** **(n. 93)**	**Total sample** **(n. 193)**	**M** **(n. 10)**	**F** **(n. 4)**	**Total sample** **(n. 14)**
**Age (years)**	64.2 ± 15.3	58.9 ± 17.9^*^	61.6 ± 16.8	71.3 ± 13.9	74.3 ± 8.9	72.1 ± 12.4^**^
**Weight (kg)**	76.6 ± 15.6	68.8 ± 16.1^*^	7 2.9 ± 16.3	63.1 ± 14.7	56.3 ± 8.3	61.2 ± 13.2^**^
**Stature (cm)**	171 ± 8	160 ± 6^*^	166 ± 9	167 ± 8	155 ± 4	163 ± 9
**BMI (kg/m** ^**2**^ **)**	26.1 ± 4.6	26.9 ± 6.3	26.5 ± 5.5	22.6 ± 3.8	23.6 ± 2.8	22.8 ± 3.4^**^
**Calf Circ. (cm)**	35.7 ± 3.9	35.4 ± 4.4^*^	35.6 ± 4.1	31.1 ± 3.9	31.8 ± 4.4	30.5 ± 5.2^**^
**Phase Angle (degrees)**	4.9 ± 1.4	4.5 ± 0.9^*^	4.7 ± 1.2	4.0 ± 1.4	3.8 ± 0.2	3.9 ± 1.3^**^
**HGS (kg)**	24.4 ± 9.4	14.2 ± 5.3	24.3 ± 9.4	19.8 ± 8.1	10.8 ± 2.9	20.5 ± 7.8^**^

* *p < 0.05 between sexes*.

** *p < 0.05 between score NRS (ALL)*.

*BMI, body mass index; HGS, handgrip strength; Calf. circ., calf circumference*.

Specifically, for patients with NRS-2002 scores ≥ 3, 14% (*n* = 2) had a BMI < 18.5 kg/m^2^, 64.3% (*n* =9) had a CC <31 cm, 79% (*n* = 11) had a PhA < 5°, and 79% (*n* =11) had a HGS < 16 for female and <27 for males.

The patients in the SGA-c group (3 M; 5 F) were older and had lower body weights than those in the SGA-b (53 M; 50 F) and the SGA-a (52 M; 44 F) groups. No significant differences were observed in height or BMI (Table 4).

**Table 4:** Anthropometric characteristics, handgrip strength and body composition according to SGA score in males, females and total sample.

	**SGA-a**			**SGA-b**			**SGA-c**		
	**M** **(n.52)**	**F** **(n. 44)**	**Total sample** **(n. 96)**	**M** **(n. 53)**	**F** **(n. 50)**	**Total sample** **(n. 103)**	**M** **(n. 5)**	**F** **(n. 3)**	**Total sample** **(n. 8)**
**Age (years)**	63.4 ± 15.6	57.3 ± 17.9	60.6 ± 16.9	65.2 ± 15.2	61.3 ± 18.2	63.3 ± 16.8	76.0 ± 7.3^*^	61.3 ± 6.5	70.5 ± 0.0^**^
**Weight (kg)**	78.3 ± 14.1	70.7 ± 14.9^*^	74.8 ± 14.9	73.6 ± 17.1	66.6 ± 16.4^*^	70.2 ± 17.1	63.9 ± 15.9	62.3 ± 25.4	63.3 ± 18.1^**^
**Stature (cm)**	173 ± 7	160 ± 6^*^	167 ± 9	169 ± 8	159 ± 7^*^	164 ± 9.0	167 ± 10^*^	159 ± 3	164 ± 9
**BMI (kg/m** ^**2**^ **)**	26.3 ± 4.4	27.3 ± 5.5	26.8 ± 5.0	25.6 ± 4.8	26.5 ± 6.7	25.9 ± 5.8	22.5 ± 3.2	24.7± 9.6	23.3 ± 5.8
**Calf. circ. (cm)**	35.9 ± 3.4	35.6 ± 3.8	35.7 ± 3.6	34.9 ± 4.7	35.3 ± 4.9	35.1 ± 4.8	32.8 ± 4.0	33.5 ± 5.8	31.8 ± 6.8^**^
**Phase Angle (degrees)**	5.5 ± 1.3	4.6 ± 0.9^*^	5.1 ± 1.2	4.3 ± 1.0	4.5 ± 0.3	4.4 ± 1.2^***^	3.5 ± 0.6	3.8 ± 1.5	3.6 ± 0.9^**^
**HGS (kg)**	26.2 ± 8.9	14.9 ± 5.8^*^	21.1 ± 9.4	22.3 ± 9.6	13.4 ± 4.7^*^	20.6 ± 8.8	18.9 ± 6.4^*^	11.0 ± 1.0	19.9 ± 6.3^***^

* *p < 0.005 between sexes*.

** *p < 0.005 vs. SGA-a and SGA-b score (ALL)*.

*** *p < 0.005 vs. SGA (a) score (ALL)*.

*BMI, body mass index; HGS, handgrip strength; Calf. circ, calf circumference*.

Both patients classified as moderate and severe malnourished according to the GLIM criteria, had significantly lower PhA and CC than not malnourished patients. Also, after adjusting the analysis by age, the differences in the results were confirmed.

Twenty-five percent of the patients (*n* = 2) in the SGA-c group had a BMI <18.5 kg/m^2^, 50% (*n* = 4)of them had a CC < 31 cm, 87.5% (*n* = 7) had a PhA < 5°, and 88% (*n* = 7= had HGS values < 16 for F and <27 for M.

In addition, SGA-c patients had significantly lower CC, HGS and PhA values than SGA-b and SGA-a patients.

When considering patients with the worst scores (i.e., SGA-c and NRS ≥3), a high prevalence of patients with low CC, PhA and HG values was observed.

About the SGA-c score, the liner regression model highlights that only PhA is an independent prognostic factor (*p* = 0.016); while, regarding to NRS-2002 ≥ 3, in the multiple regression analysis, the presence of a low calf circumference (*p* = 0.015) was the major independent predictor.

## Discussion

The prevalence of malnutrition among hospitalized patients has been widely documented in the literature and is estimated to be between 20% and 50%, depending on the patient population and criteria used for diagnosis.

In this preliminary study, nutritional risk was assessed by the NRS-2002 screening test, the SGA and the GLIM criteria in a heterogeneous sample of Caucasian patients, hospitalized in either medical or surgical units following visits to the emergency department.

According to the NRS-2002, 7% of the patients were at high nutritional risk or malnourished (score ≥3), while according to the SGA, 49.8% of patients were moderately (SGA-b) and 3.9% (SGA-c) severely malnourished. Based on the GLIM criteria, when using all combinations of the two-criteria diagnosis identified about 18% of patients to be malnourished. When severe malnutrition was considered, GLIM identified a higher proportion (around 51%) than SGA (around 4%). Based on prevalence alone and compared to SGA, GLIM seems to represent a larger proportion of overall malnutrition but is more likely to identify a person as severely malnourished.

This apparent inconsistency in results may be primarily because the SGA classified patients into one of three levels: (a well-nourished, b suspected malnutrition or moderate malnutrition, c severely malnourished), whereas the NRS 2002 addresses two categories only (no nutritional risk or severe malnutrition). Although both the NRS 2002 and the SGA consider the metabolic stress of disease and changes in food intake, the NRS-2002 classifies metabolic stress using numerical scores, while the SGA depends on the investigator's experience to indicate the metabolic stress of disease (28).

Moreover, the NRS-2002 contains questions that indicate recent or acute changes in nutritional status (percent of weight loss in the last three months) and age, while the SGA includes questions related to the detection of chronic malnutrition (such as percent of weight loss in the last six months, change in consistency of the diet, presence of gastrointestinal symptoms, loss of subcutaneous fat, and the presence of oedema). This variety of questions could also be responsible for the different results (6, 30–32).

The NRS-2002 is a fast, easy and useful screening tool that seems well-suited to be applied in an acute phase to patients coming from an emergency department; however, the exclusive use of the NRS-2002 might underestimate the real incidence of malnutrition in hospitals (6).

Soon after patient stabilization, the SGA could be integrated into nutritional evaluations. In September 2018, in order to build a global consensus on the diagnostic criteria for malnutrition, the GLIM criteria were proposed. According to these criteria, patients were categorized into two groups: moderate (stage 1) and severe (stage 2) malnutrition. This study showed that both patients with moderate and severe malnutrition had lower PhA and CC compared with those without malnutrition.

At our best knowledge, the GLIM criteria had been still not validated in patients coming from an Emergency Department.

PhA is the most clinically established impedance parameter and has been suggested to be an indicator of cellular health, and nutritional status, and be highly predictive of impaired clinical outcome and mortality in a variety of disease.

PhA represent a clinically feasible approach to body composition, free from equation inherent errors and necessary assumption. It been shown to be a superior indicator of survival and outcome and is generally used as screening tool for identification of patients at nutritional risk. BIVA provides more detailed information on hydration and cell mass integrity and should be considered as an assessment and monitoring tool (13).

Low PhA values predict poor outcomes, long hospital stays and morbidities (16, 33). In our study, PhA values < 5° were observed in 93% of the patients with NRS scores ≥3 and in 88% of patients in the SGA-c group.

HGS seems to detect muscle loss, fiber quality, and functionality earlier while providing a better evaluation of nutrition repletion after therapy (34). It may be considered a functional and nutritional indicator that adequately predicts hospitalization costs (17). Several studies have shown that HGS is both sensitive and specific in predicting increased postoperative complications and is associated with longer length of hospital stay and long-term mortality among hospitalized patients (35, 36).

In our study, very low HGS values (<16 in F and <27 in M) were found in patients with SGA-c scores and in those with an NRS-2002 ≥ 3; this association has also been confirmed by several studies showing lower HGS among patients at high nutritional risk, as evaluated by both screening tests (36, 37).

In summary, in our study, low SGA (SGA-c) scores were clearly associated with the lowest values of PhA, while CC (p.0.015) has the major prognostic role in patients with NRS 2002 ≥3, and as for the GLIM criteria the same happens with the lowest PhA values (p. 000) and CC.

Low PhA and HGS values were observed in all Caucasian hospitalized patients evaluated, independent of their SGA and NRS-2002 and GLIM scores. These results confirm the need for close monitoring of all patients during hospitalization (including those with initially good screening tests) to detect possible changes in clinical and nutritional status early on.

## Limitations Of The Study

Study limitations are the relatively heterogeneous population, the small sample size, the data results from a single hospital, the lack of detailed clinical information on the evaluated patients and of their clinical outcomes; these last two deficiencies are linked to the nature of the observational study. Another study limitation is that we have not used BIVA to better define if low PhA was linked to malnutrition, fluid overload or both. Anyway, this study had not the primary aim to obtain detailed information on hydration.

## Data Availability Statement

The raw data supporting the conclusions of this article will be made available by the authors, without undue reservation.

## Ethics Statement

The studies involving human participants were reviewed and approved by Ethical Committee of the Federico II University Hospital. The patients/participants provided their written informed consent to participate in this study.

## Author Contributions

FP, GD, and FC conceived and designed the study. ES, OD, CD, and MN collected research data. OD, DM, and MM analyzed the results. ES and MM drafted the manuscript. LS and FP made critical revisions to the manuscript for important intellectual content. All authors have read and approved the final manuscript.

## Conflict of Interest

The authors declare that the research was conducted in the absence of any commercial or financial relationships that could be construed as a potential conflict of interest.

## Publisher's Note

All claims expressed in this article are solely those of the authors and do not necessarily represent those of their affiliated organizations, or those of the publisher, the editors and the reviewers. Any product that may be evaluated in this article, or claim that may be made by its manufacturer, is not guaranteed or endorsed by the publisher.
