# Supplementary material for: Nutritional Screening and Anthropometry in Patients Admitted From the Emergency Department
Source: Front Nutr. 2022 Feb 14;9:816167. doi: 10.3389/fnut.2022.816167 (PMC8882738; doi:10.3389/fnut.2022.816167)
Supplement: Supplementary file 1 [file Table_1.docx]

**Table1: Regression analysis models predicting the SGA-c score**

|  | **OR** | **95% Confidence Interval** | **p** |
| --- | --- | --- | --- |
| **PhA** | 0.390 | 0.181-0.841 | 0.016 |
| **Calf circ.** | 0.863 | 0.714-1.043 | 0.128 |
| **BMI** | 0.878 | 0.744-1.035 | 0.121 |
| **HG** | 0.952 | 0.869-1.043 | 0.293 |
| **SEX** | 1.506 | 0.350-6.475 |  |
| **AGE** | 1.041 | 0.983-1.103 | 0.582 |

*Calf circ. calf circumference; HG, Handgrip strenght*

**Table2: Regression analysis models predicting the NRS-2002 ≥3 score**

|  | **Univariate analysis** | |  | **Multivariate analysis** | | |
| --- | --- | --- | --- | --- | --- | --- |
|  | **OR** | **95%**  **Confidence Interval** | **p** | **OR** | **95%**  **Confidence Interval** | **p** |
| **PhA** | 0.530 | 0.311-0.906 | 0.020 | 0.626 | 0.343-1.145 | 0.128 |
| **Calf circ.** | 0.724 | 0.607-0.864 | 0.000 | 0.766 | 0.618-0.950 | 0.015 |
| **BMI** | 0.847 | 0.741-0.969 | 0.015 | 0.969 | 0.829-1.131 | 0.688 |
| **HG** | 0.972 | 0.911-1.037 | 0.392 |  |  |  |
| **SEX** | 2.348 | 0.712-7.747 | 0.161 |  |  |  |

*Calf circ. calf circumference; HG, Handgrip strenght*
